# CAREGIVING STRESSORS AND MENTAL HEALTH AMONG OLDER DEMENTIA CAREGIVERS: THE MEDIATING ROLE OF LIFE DISRUPTION

**DOI:** 10.1093/geroni/igac059.2203

**Published:** 2022-12-20

**Authors:** Fei Wang, Elliane Irani

**Affiliations:** Case Western Reserve University, Cleveland, Ohio, United States; Case Western Reserve University, Cleveland, Ohio, United States

## Abstract

Providing care to a loved one with dementia can take a mental toll on older adult caregivers who struggle to balance their caregiving duties and self-care. However, few caregiving studies focus on older adults as caregivers. Further, the extant research mainly investigates stressors directly related to the demands of caregiving without accounting for the disruptions in different areas of life triggered by dementia caregiving. Guided by the Stress Process Model, we examined how life disruption mediates the association between caregiving stressors and caregiver mental health. A total of 360 older adult caregivers (age 65 or above) of individuals with dementia (age 65 or above) in the U.S. were selected from the 2017 National Study of Caregiving. An index of life disruption was created based on seven types of life challenges due to caregiving, such as constriction of social life and job-caregiving conflict. Results from a path analysis show that both objective and subjective caregiving stressors (i.e., care assistance and role overload) were positively associated with life disruption, which in turn, were positively associated with depressive symptoms and anxiety. Caregiver socio-demographics and self-rated health were included in the analysis as covariates. These findings inform a novel clinical assessment framework for practitioners to identify the unique challenges faced by older dementia caregivers in the U.S. Moreover, this study elucidated life disruption as a modifiable factor to mitigate mental health problems in this population. Future research should compare the effects of different types of life disruption on mental health among older dementia caregivers.

